# A Real-Time Apple Grading System Using Multicolor Space

**DOI:** 10.1155/2014/292681

**Published:** 2014-01-19

**Authors:** Hayrettin Toylan, Hilmi Kuscu

**Affiliations:** ^1^Pinarhisar Vocational High School, Department of Electricity, Kırklareli University, 39300 Kırklareli, Turkey; ^2^Department of Mechanical Engineering, Faculty of Architecture and Engineering, Trakya University, 22030 Edirne, Turkey

## Abstract

This study was focused on the multicolor space which provides a better specification of the color and size of the apple in an image. In the study, a real-time machine vision system classifying apples into four categories with respect to color and size was designed. In the analysis, different color spaces were used. As a result, 97% identification success for the red fields of the apple was obtained depending on the values of the parameter “*a*” of CIE L*a*b*color space. Similarly, 94% identification success for the yellow fields was obtained depending on the values of the parameter *y* of CIE *XYZ* color space. With the designed system, three kinds of apples (Golden, Starking, and Jonagold) were investigated by classifying them into four groups with respect to two parameters, color and size. Finally, 99% success rate was achieved in the analyses conducted for 595 apples.

## 1. Introduction

Turkey has the third place in the world after China and the USA with a 2.782.370-ton apple production [1]. The apples produced are demanded by the customers depending on their different features like type, color, size, and defects. However, one of the most important things in apple marketing is standardization. In the past, color and size classification of the apples were done by employees by just looking at them. It is hard to classify apples that are in similar colors yet in different types. However, due to the different perceptions of the employees, classification mistakes were being made. Most of the fruit classification systems built in Turkey classify fruits according to their size and mass. Machines based on mass of fruit need a mass measurement system. This type of machines can be efficiently used for 2000 kg/h. Different shape of circular materials or holey wooden staffs are used for eliminating the fruits according to their size. By using these machines a person can classify 100–200 fruits per hour. Even if centrifugal technology using machines are commonly used in our country [2], the leading actors of the sector think that it is not good enough for latest business conditions.

Recently, with the improvement of vision processing technology, machine vision has started to be used in industrial food machines which enable the classification of different types of fruits in terms of their size, color, and defects with help of high-definition cameras. Machine vision systems or vision processing techniques have been used by researchers on different types of fruits [3], such as citrus [3], cherries [4], peach [5], olive [6, 7], orange [8], pistachio [9], mushroom [10], and apple [11–14].

Leemans and Destain obtained the RGB images of Jonagold variety of apples via two CCD cameras placed in different angles. By using the images obtained, they were able to classify the apples into two quality groups with a 73% success rate by the segmentation of the apple surface [11]. Li et al. placed two CCD cameras and two mirrors in such a way that the top and bottom surfaces of the Fuji variety of apples can be seen. They segmented the apple images by the developed algorithm, placing an 840 nm band-pass filter to the camera lenses. Their results show that segmentation and classification processes for an apple are 320 ms [12]. Wen and Tao obtained the images of red delicious variety of apples by using one CCD camera and a 700 nm long-pass filter on the lens. They also performed a fast blob extraction on the images by an adaptive spherical transformation. Out of total 960 samples, these samples consist of 232 good and 728 defective apples. The success rate for the identification of the good quality apples is 93.7% while it is 95.88% for defective apples [14]. Kavdir and Guyer achieved the classification of golden delicious variety of apples into three categories by using three different methods: nearest neighbor, decision tree classifier, and multilayer perception. The highest success rate with 90% was obtained by multilayer perception method [13]. In the aforementioned studies, it was focused on the defect classifications of the apples and segmentation of the apple images obtained. On the other hand, three different varieties of apples were classified into categories according to their color and size in this study. The RGB images of apples were tested in different color spaces. It was tried to obtain the color and size of the apples with the highest possible speed and accuracy. The success in this process is dependent on the right selection of the color space and their transformations.

Color is the main information source for object classification, inspection, and sorting. Color space is the mathematical representation of colors. Color spaces are formed so that they represent all colors. Color spaces are designed three dimensionally. The most frequently used color spaces are RGB (on computer monitors), YIQ, YUV or YCbCr (in video systems), and CMYK (in color printers). Since none of these color spaces are related to brightness, saturation, and intuitive perception of the color, there are additional color spaces such as HSV and HSI [15]. Color spaces can be grouped under two titles. In device dependent color spaces, features of the machine are particularly important. The device independent color spaces are color spaces developed by Commission Internationale de L.Eclairage (CIE). CIE *XYZ*, CIE L*a*b*, and CIE L*C*h* can be counted among device independent color spaces.

There have been several benefits of applying color space transformation that have been identified by studies on skin detection. First, the codification process is sharpened to enhance the distinctiveness between skin and nonskin classes through a certain color space transformation. Another benefit is the realization of illumination invariance, as varying illumination has its own set of challenges regarding skin detection. Finally, color space transformation presumably is able to categorize shades of different skin tones together [16]. So, HSV color spaces are used for skin color detection by Chaves-González et al. [17]. Also CIE L*a*b* system is suggested as the best color space for quantification in foods with curved surfaces [18] and when HSV color space transformations are combined with Support Vector Machine (SVM) classifier, 96.7% success is obtained in classification of pizza toppings [19].

There is a lot of studies in the literature on the classification of apple. In this study, with the difference of other studies in the literature, a conveyor system was developed to obtain data from different angles of each apple. The apple image was separated from the background by HSV color space component with thresholding. Apple size and color were obtained by multicolor space (HSV, CIE L*a*b*, and CIE *XYZ* color space) component with thresholding. Different color space transformations were investigated to find the best color space in the classification of apples.

## 2. Materials and Methods

### 2.1. Machine Vision System Hardware

**Figure 1:**
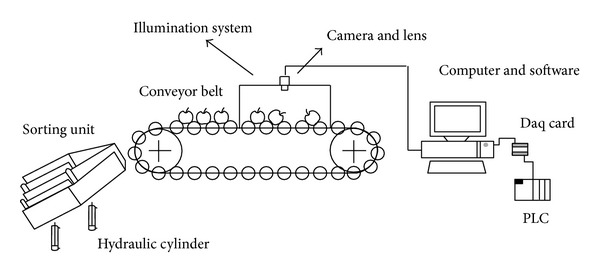
Schematic representation of machine vision system.

**Figure 2:**
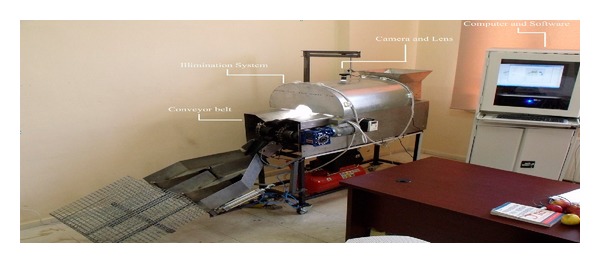
Machine vision system.

Image acquisition device used for this research is a 1.3 mega pixel (*H* × *V* = 1280 × 1024 pixel) resolution 25 fps (frame per second) Complementary Metal Oxide Semiconductor (CMOS) camera with a manually adjustable 6 mm focus length which is mounted on camera, C-mount lens, a lighting system, 600 mm × 670 mm × 300 mm white painted diffusely illuminated tunnel with four fluorescent lamps, a conveyor belt on which fruits are placed, and automatic sorting unit. The setup of the system is shown in Figures 1 and 2. The algorithms used were developed with Matlab.

**Figure 3:**
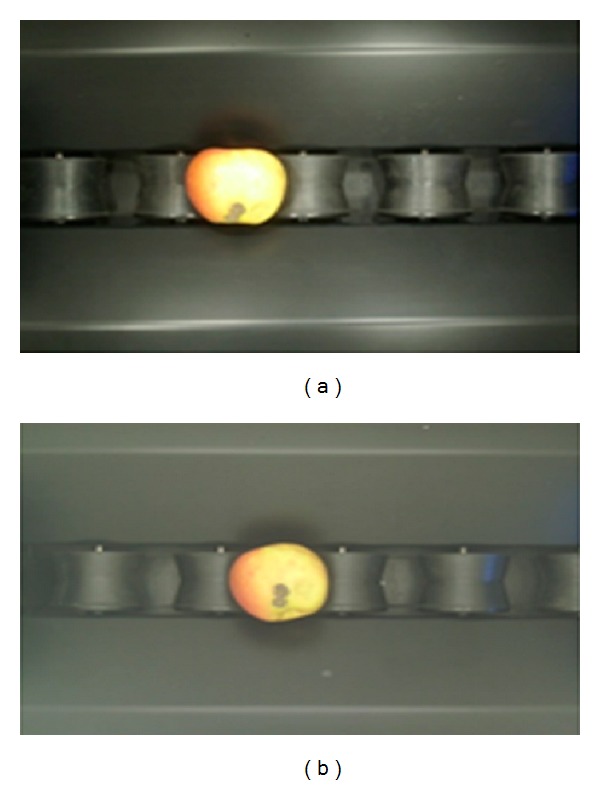
Direct illuminated apple image with (a) halogen lamps, (b) fluorescent lamps.

**Figure 4:**
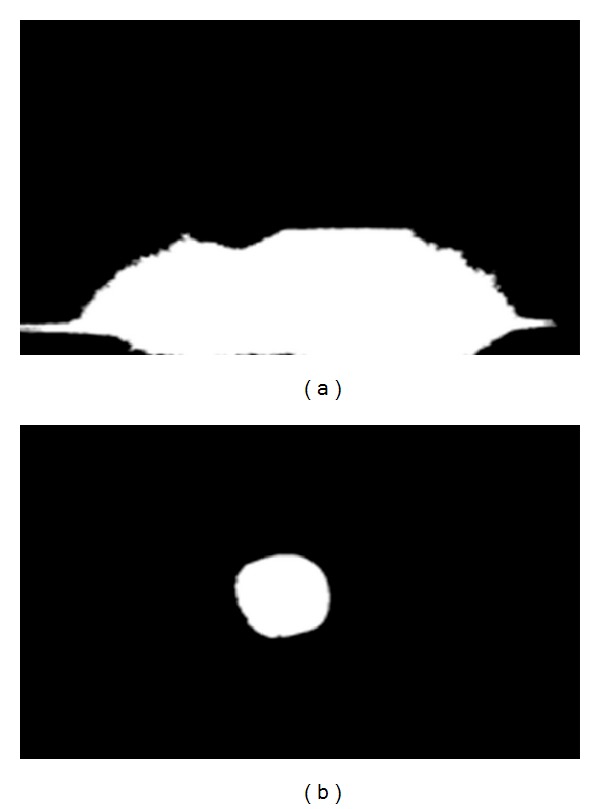
The masking results of illuminated apple image with (a) halogen lamps, (b) fluorescent lamps.

Machine vision systems are affected by the level and quality of illumination [20]. The changes in daylight level, wrong positioning of the lamp, together with the wrong choice of lamp could have negative effects on the software used in the system. Images of object illuminated with halogen lamps and fluorescent lamps are shown in Figures 3(a) and 3(b). Masking results of object illuminated with halogen lamps and fluorescent lamps are shown in Figures 4(a) and 4(b). The transition functions among the color spaces used in the software change depending on the illumination characteristic of the lamp. The space where images are collected in the machine vision system is separated from natural lighting. In this area, artificial lighting is used.

In practice, the points to be taken into consideration in the design of lighting systems are color rendering, high index, close to daylight (color temperature 5000 K−6500 K), choice of lamp whose level of light is appropriate, and proper positioning of the lamp so that all surfaces of the object receive equal levels of light. In the lighting system, 4 Philips TL-D 90 Graphica 18W/950 models are used. The features of this lamp are a color temperature of 5300 K (approximately D55) and a color-rendering index (*R*_*a*_) close to 97%.

### 2.2. Color Spaces

Generally, color images are taken by a color camera and saved in the three-dimensional RGB (red, green, and blue) color space. Unfortunately, RGB color space is not consistently uniform, and the proximity of colors does not indicate color similarity [21]. In addition, different color space transformation may be powerful tool for object recognition.

#### 2.2.1. RGB Color Space

An RGB color image is an *M* × *N* × 3 array of color space pixels, where each color pixel is a triplet corresponding to the red, green, and blue components of an RGB image at a specific spatial location [22].

*R*,  *G*, and  *B*  values of RGB color space are the sum of respective sensitivity functions and the incoming light;

(1) $$\begin{array}{c} R=\int_{300}^{830}S\left(\lambda \right)R\left(\lambda \right)d\lambda ,\\ G=\int_{300}^{830}S\left(\lambda \right)G\left(\lambda \right)d\lambda ,\\ B=\int_{300}^{830}S\left(\lambda \right)B\left(\lambda \right)d\lambda , \end{array}$$

where  *S*(*λ*)  is the light spectrum, *R*(*λ*), *G*(*λ*), and *B*(*λ*) are the sensitivity functions for the *R*, *G*, and *B* sensors, respectively [23].

#### 2.2.2. HSV Color Space

HSV color space is quite closer than the RGB system to the way in which human experiences and describes color sensations [22]. HSV separates color into three components: hue, saturation, and value. *H* (hue) distinguishes among the perceived colors, such as red, yellow, green, and blue.  *V* (value) represents the brightness of a color and *S* (saturation) refers to how much the amount of white light mixed with a hue is [21]. Consider the following.

HSV color space values are calculated according to *R*, *G*, and *B* values [22]

(2) $$\begin{array}{c} H=\{\begin{array}{cc} \theta , & \text{if}\;\;B\leq G,\\ 360-\theta , & \text{if}\;\;B>G, \end{array}\\ \theta =\cos^{-1}\left(\frac{0.5\left(R-G\right)+\left(R-B\right)}{\sqrt{{\left(R-G\right)}^{2}+\left(R-B\right)\left(G-B\right)}}\right),\\ S=1-\left(\frac{3}{R+G+B}\right)\min\left(R,G,B\right),\\ \text{Value}\left(V\right)=\max\left(R,G,B\right). \end{array}$$

#### 2.2.3. CIE *XYZ* Color Space

Since CIE *XYZ* color space takes the human eye as a basis, it forms the base for all color management systems and includes all perceivable colors. This color space is determined by CIE in 1931 according to standard illuminant (A, B, C, D50, D55, D65, E, and F) and standard observer (2°, 10°). The reason for this is that the most significant feature of CIE *XYZ* color space is being device independent.

Tristimulus values at wavelength *λ* of CIE color system [24] are

(3) $$\begin{array}{c} X=k\int^{}S\left(\lambda \right)P\left(\lambda \right)x\left(\lambda \right)d\lambda ,\\ Y=k\int^{}S\left(\lambda \right)P\left(\lambda \right)y\left(\lambda \right)d\lambda ,\\ Z=k\int^{}S\left(\lambda \right)P\left(\lambda \right)z\left(\lambda \right)d\lambda , \end{array}$$

where *S*(*λ*) is the relative spectral power of an illuminant, *x*(*λ*), *y*(*λ*), and *z*(*λ*) are color-matching functions, *P*(*λ*) is the spectral reflectance at wavelength *λ*, and *k* is a normalizing factor given by 100/∫*S*(*λ*)*y*(*λ*)*d*(*λ*).

#### 2.2.4. CIE L*a*b* Color Space

In 1976, CIE suggested two color spaces (CIE L*a*b* and CIE L*u*v*) in order to provide the structural equity space. There is a strong relation between these color spaces and a human's visual perception. The components are “*L*” (light), the rate from green to red “*a*”, and that from blue to yellow “*b*”.

Finding out the color components of CIE L*a*b* color space is possible with CIE *XYZ* color space. The observer and the standard illuminator used to discover *X*, *Y*, and *Z* values affect these values directly.

Transformation from CIE *XYZ* to CIE L*a*b* can be achieved by the following equations [18]:

(2.2.4) $$\begin{array}{c} L^{*}=116f{\left(\frac{Y}{Y_{n}}\right)}^{1/3}-16,\\ a^{*}=500\left[f{\left(\frac{X}{X_{n}}\right)}^{1/3}-f{\left(\frac{Y}{Y_{n}}\right)}^{1/3}\right],\\ b^{*}=200\left[f{\left(\frac{Y}{Y_{n}}\right)}^{1/3}-f{\left(\frac{Z}{Z_{n}}\right)}^{1/3}\right],\\ f\left(q\right)=\{\begin{array}{cc} q, & \text{if}\;\;q>0.008856,\\ 7.787q+\frac{16}{116}, & \text{else}; \end{array} \end{array}$$

*q* ∈ {*X*/*X*_*n*_, *Y*/*Y*_*n*_, *Z*/*Z*_*n*_}, *X*_*n*_,  *Y*_*n*_, and *Z*_*n*_ are the tristimulus values of the illuminant, in this case illuminant is D55.

### 2.3. Image Background Removal via Different Color Spaces

Apple area was separated from the background with thresholding. The RGB image is transformed into HSV with the “rgb2hsv” routine of the image processing toolbox of Matlab (Mathworks 2011) before applying thresholding. In addition, the RGB image is transformed to CIE *XYZ* and CIE L*a*b* with D55-compliant. The sorting process is carried out by collecting the thresholding results of HSV color space “*S*” component, CIE *XYZ* color space “*Y*” component, and CIE L*a*b* “*a*” component in one mask. Consider the following:

(5) $$\begin{array}{c} f\left(x,y\right)=\{\begin{array}{cc} 1, & \text{if}\;\;z\left(x,y\right)>T,\\ 0, & \text{else}, \end{array} \end{array}$$

where *f*(*x*, *y*) is the image obtained after the background has been removed, with *z*(*x*, *y*) being the original image and *T* being the threshold. The threshold values based on image. The threshold values are defined by examining the histograms of the color space components. These values are fixed and added to the software after testing different apple images.

### 2.4. Identification of Apple Color and Size

The next process after the process of separation of the background is the determination of the color and size of the apple. Size of the apple is calculated according the pixel numbers it occupies in *x*- and *y*-axes in the masked area.

The color of the apple is determined according to the intensity and dispersion of the colors in the specified area. For this procedure, thresholding is used for red and yellow regions in the image. The thresholding of “*Y*” component of CIE *XYZ* color space shows the yellow regions in the apple picture. The thresholding of “*a*” component of CIE L*a*b* color space shows the red regions in the apple picture. The information about the color and the type of the apple is gathered according to the area the yellow and red regions take up on the surface of the apple. The conversion of RGB apple picture to CIE *XYZ* and CIE L*a*b* color spaces can be applied with the help of built-in functions of Matlab. Yet, the conversion time of Matlab built-in function is too much for a real-time system. However, only one component of these color spaces is used.

### 2.5. RGB to CIE *XYZ* Conversion

The conversion from RGB color space to CIE *XYZ* and CIE L*a*b* color spaces changes depending on the illuminant character of the lamp. For this reason, a function is written considering that the color temperature of the fluorescent lamp is 5300 K (approximately D55).

Firstly, nonlinear *R*, *G*, and *B* values are converted to linear and nominal values as follows:

 
*R*′ = *R*/255 , *G*′ = *G*/255, *B*′ = *B*/255
 If *R*′, *G*′, *B*′ ≤ 0.04045 (IEC 61966-2-1 std, 1999)
 
*R*_*s*_ = *R*′/12.92;  *G*_*s*_ = *G*′/12.92;  *B*_*s*_ = *B*′/12.92 Else
  (6) $$\begin{array}{c} Rs={\left[\frac{R^{\prime }+0.055}{1.055}\right]}^{2.4};\;\;Gs={\left[\frac{G^{\prime }+0.055}{1.055}\right]}^{2.4};\\ Bs={\left[\frac{B^{\prime }+0.055}{1.055}\right]}^{2.4}. \end{array}$$

Converting RGB to CIE *XYZ* is performed with the help of a 3 × 3 matrix.

(7) $$\begin{array}{c} \left[\begin{array}{c} X\\ Y\\ Z \end{array}\right]=\left[M\right]\left[\begin{array}{c} R_{S}\\ G_{S}\\ B_{S} \end{array}\right]. \end{array}$$

The conversion from reference white RGB color space to average daylight (D65), CIE *XYZ*_D65_ color space is given below [25];

(8) $$\begin{array}{c} \left[\begin{array}{c} X\\ Y\\ Z \end{array}\right]=\left[\begin{array}{ccc} 0.4124564 & 0.3575761 & 0.1804375\\ 0.2126729 & 0.7151522 & 0.0721750\\ 0.0193339 & 0.1191920 & 0.9503041 \end{array}\right]\left[\begin{array}{c} R\\ G\\ B \end{array}\right]. \end{array}$$

### 2.6. From *XYZ*_D65_ (Source Illuminated) to *XYZ*_D55_ (Destination Illuminated) Transform

If the source differs from the illuminant of the destination, chromatic adaptations are used to decide whether the *XYZ* source data differs from the illuminant target RGB space (destination). Humans have a visual ability, called chromatic adaptation, to acknowledge and discount the color of the illumination and approximately recall an object's color. Chromatic adaptation is most easily understood by viewing a white object under various illuminations. Several chromatic adaptation transformations exist in literature, most of which are based on the von Kries model [26, 27].

By using von Kries transforms, *XYZ*_D65_ color space acquired according to D65 reference white is converted to CIE *XYZ*_D55_ color space according to the D55 reference white used in our lighting system. Consider the following:

(9) $$\begin{array}{c} \left[\begin{array}{c} X_{D}\\ Y_{D}\\ Z_{D} \end{array}\right]=\left[M\right]\left[\begin{array}{c} X_{S}\\ Y_{S}\\ Z_{S} \end{array}\right],\\ \left[M\right]={\left[M_{A}\right]}^{-1}\left[\begin{array}{ccc} \frac{\rho_{D}}{\rho_{S}} & 0 & 0\\ 0 & \frac{\gamma_{D}}{\gamma_{S}} & 0\\ 0 & 0 & \frac{\beta_{D}}{\beta_{S}} \end{array}\right]\left[M_{A}\right],\\ \left[\begin{array}{c} \rho_{S}\\ \gamma_{S}\\ \beta_{S} \end{array}\right]=\left[M\right]\left[\begin{array}{c} X_{WS}\\ Y_{WS}\\ Z_{WS} \end{array}\right],\;\;\left[\begin{array}{c} \rho_{D}\\ \gamma_{D}\\ \beta_{D} \end{array}\right]=\left[M\right]\left[\begin{array}{c} X_{WD}\\ Y_{WD}\\ Z_{WD} \end{array}\right]. \end{array}$$

*X*_*WS*_,  *Y*_*WS*_, and  *Z*_*WS*_  are source reference white;  *X*_*WD*_,  *Y*_*WD*_, and  *Z*_*WD*_  are the destination reference white;  *ρ*,  *γ*, and  *β*  are cone response domain. Accordig to conditions of D65 luminance, source reference values are  *X*_*WS*_,  *Y*_*WS*_,  *Z*_*WS*_ = 0.9505, 1.0000, 1.0890 respectively. Accordig to conditions of D55 luminance, destinations reference values are  *X*_*WD*_,  *Y*_*WD*_,  *Z*_*WD*_ = 0.95682, 1.0000, 0.92149 respectively. In calculations we assumed the 2° standard colorimetric observer. The conversion matrix acquired via chromatic adaptation matrix (*M*_*A*_) is

(10) $$\begin{array}{c} \left[M\right]=\left[\begin{array}{ccc} 1.0096 & 0.0331 & -0.0330\\ 0.0036 & 0.9973 & -0.0007\\ 0 & 0 & 0.8463 \end{array}\right]. \end{array}$$

## 3. Results and Discussion

Using the designed system, ten images were obtained for the test purpose. On each image, there is one yellow and one red apple. The experimental results for the average and standard deviation values of the parameters of different color spaces for the color pixels of red and yellow apples and the background are given in Table 1. These values were calculated considering 5000 pixels for each field.

The apple images are obtained with special fluorescent lamps which have a color temperature of 5300 K (approximately D55) and a color-rendering index (*R*_*a*_) that is close to 97%. These RGB images are converted to the CIE *XYZ*_D65_ color space according to illumination condition and 2° standard observers. It is converted to the CIE *XYZ*_D55_ color space by taking into consideration the D55 illumination conditions in the systems using the von Kries transforms matrix. The CIE L*a*b* color space and components are calculated from the CIE *XYZ*_D55_ color space by using the aforementioned formulas.

After examining the histograms of the pixels along with the color parameter values (Table 1), the parameter “*a*” of the CIE L*a*b* color space was selected for the identification of the red regions. As for the identification of the yellow regions, the parameter “*Y*” of the CIE *XYZ* color space was selected. The threshold values of the selected color space were specified in accordance with the characteristics of the component. The negative values of  a*  component of CIE Lab color space indicate “green” while the positive values indicate “magenta” (close to red). Therefore, the threshold value was specified as 15. The *Y* component of CIE *XYZ* was used to expose the yellow regions by prioritizing the luminance. The threshold value was specified as 0.5 in accordance with the normalized values. Further the pixels smaller threshold values were deleted. The results show that the identification success rates for the pixels in red and yellow regions are 97% and 94%, respectively. They also show that the parameter “*S*” of the HSV color space is a useful color space to distinguish the background and to remove the apple masks.

**Figure 5:**
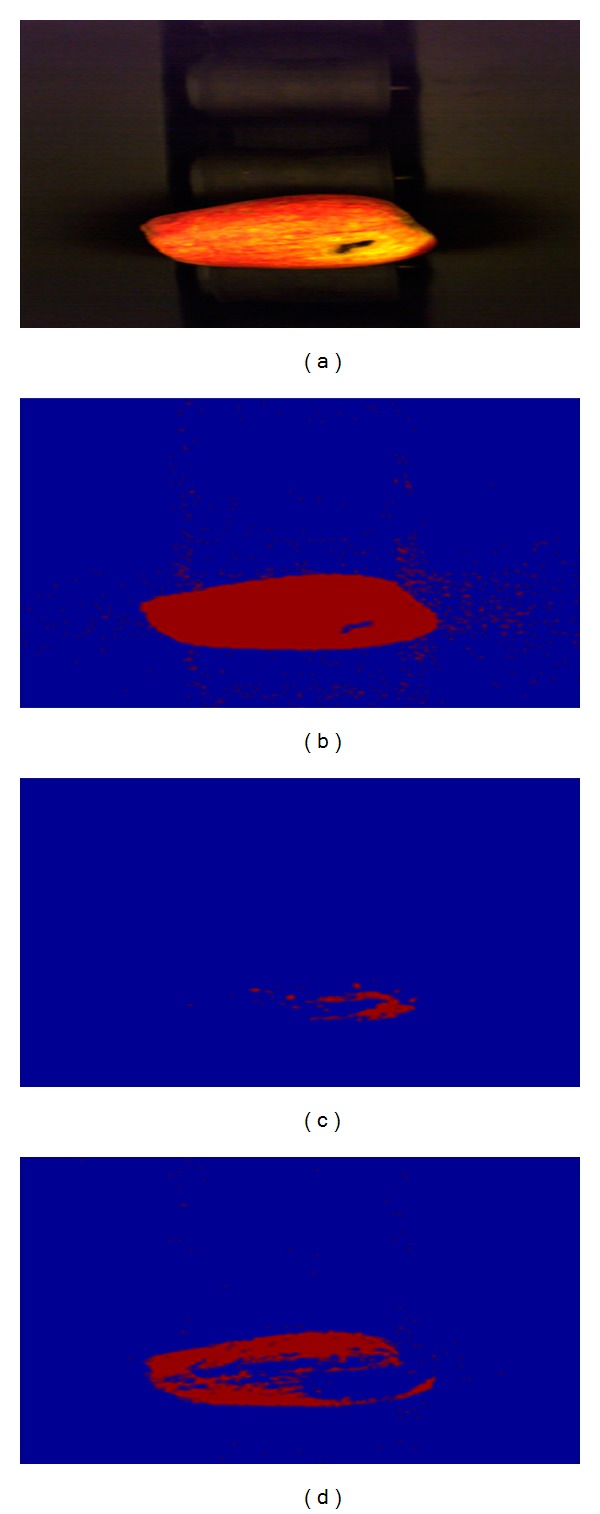
(a) Original image. Thresholding results of the (b) “*S*” component of HSV color space, (c) “*Y*” component of CIE *XYZ* color space, and (d) “*a*” component of CIE L*a*b* color space.

The size and color information is collected from the pictures acquired by the machine vision system after the place of the apple is masked in the picture. The components of color spaces used to achieve this are shown in Figure 5.

**Figure 6:**
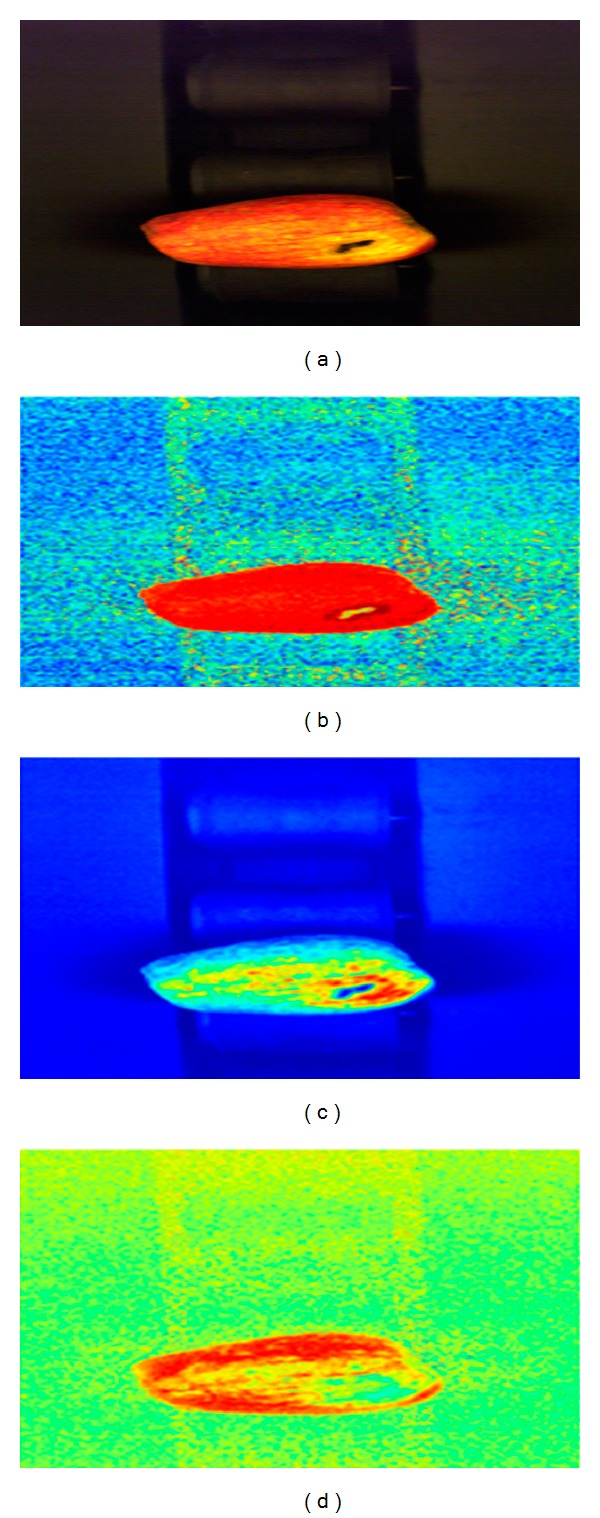
(a) Original image. Image of the (b) “*S*” component of HSV color spaces, (c) “*Y*” component of CIE *XYZ* color space, and (d) “*a*” component of CIE L*a*b* color spaces.

**Figure 7:**
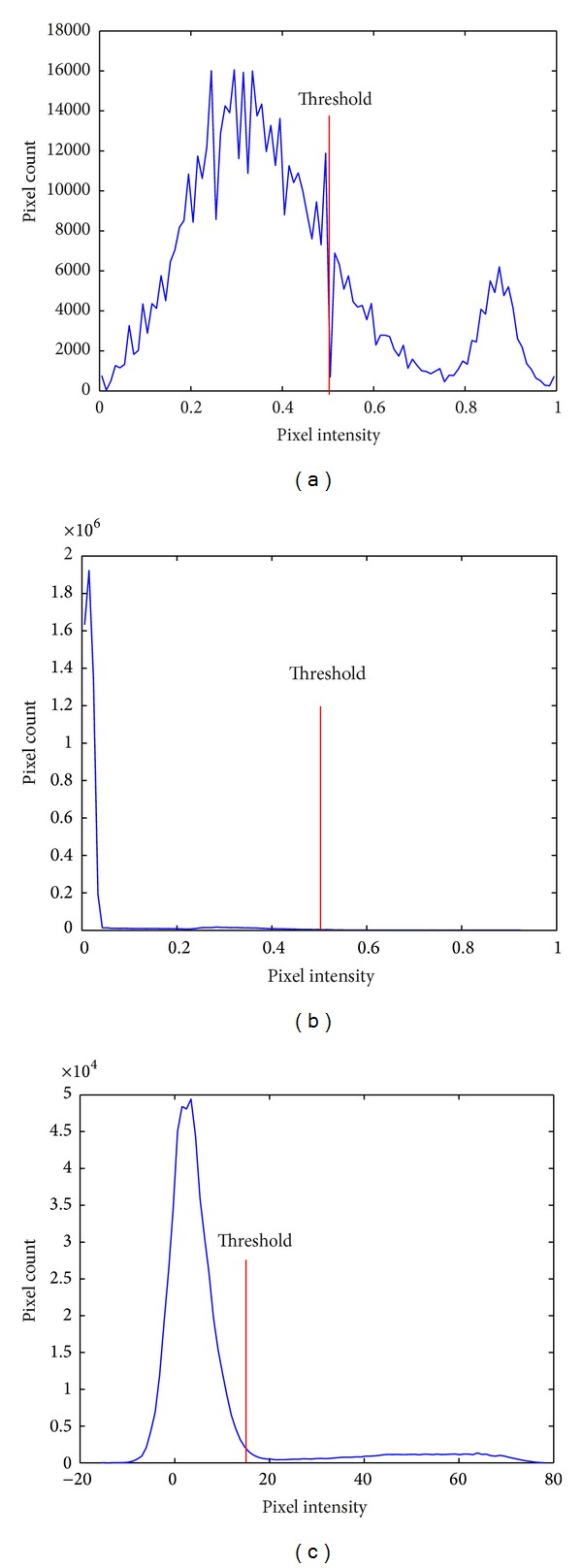
Histogram of the (a) “*S*” component of HSV color spaces, (b) “*Y*” component of CIE *XYZ* color space, and (c) “*a*” component of CIE L*a*b* color spaces.

These results show that the apple size and color were basically extracted. Multicolor space is used to separate the apple from the background and to effectively determine its color and size. The determination of the position of the apple in the image is achieved considering all the thresholding results (Figures 5(a), 5(b), and 5(c)) of the “*S*” component (Figure 6(a)) in HSV color space, “*Y*” component (Figure 6(b)) in CIE *XYZ*, and “*a*” component (Figure 6(c)) in CIE L*a*b* color space in one mask. Threshold value is obtained using color spaces components of histograms. “*S*” component histogram of HSV color space, “*Y*” component histogram of *XYZ* color space, “*a*” component histogram of CIE L*a*b* color space are presented in Figures 7(a), 7(b), and 7(c), respectively. Using the thresholding results of “*Y*” component (of CIE *XYZ* color space) and “*a*” component (of CIE L*a*b* color space), yellow and red regions are determined. Hence, apple type is obtained according to these color information.

**Figure 8:**
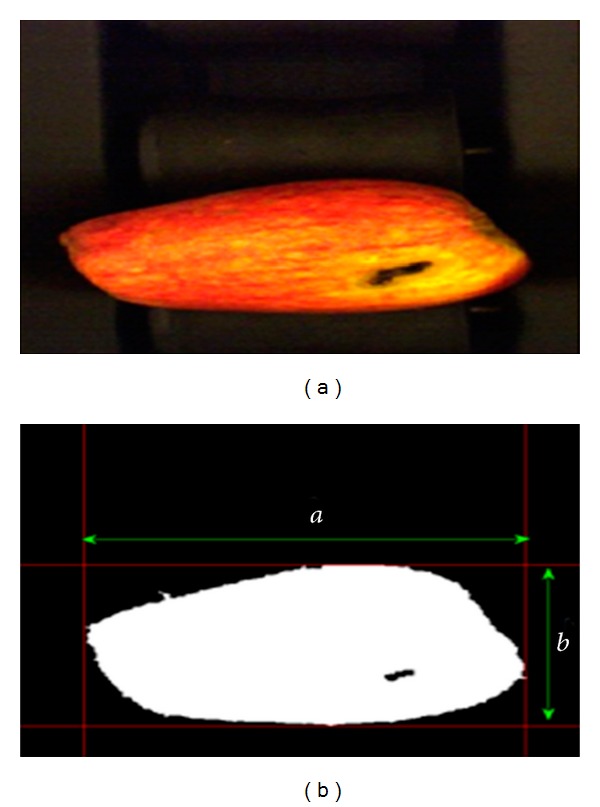
Original apple image (a) and masked image (b).

There are no red regions on the surface of the Golden apples. Note that, when there is no red region on surface of an apple, it is classified as Golden. On the other hand, if the apple surface has only red regions or has red region and yellow region together, the apples are classified differently as Jonagold or Starking. These types of apples are determined depending on the saturation and amount of the red and yellow regions. But the color (red-yellow) and size (regular-large) are taken into consideration in the experimental results. Furthermore, the size of the apple is calculated by multiplying pixel number of the area (the apple covers) in *x*- and *y*-axes in the masked area, and the pixel length (0.312 mm) (Figure 8).

**Figure 9:**
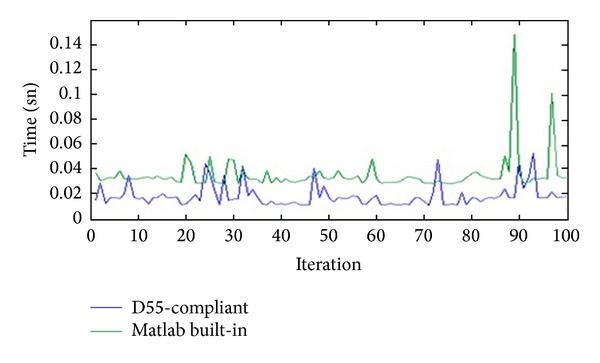
Comparison of processing time at Matlab built-in and D55-compliant software at 100 iterations.

Moreover, for device independent color space conversions, a custom, D55-compliant function is developed and runs faster than the Matlab built-in counterpart. The RGB to CIE L*a*b* conversion took on average of 34.2 ms with Matlab's built-in rgb2lab function, while the same conversion took on average of 13.4 ms with the new function (D55-compliant). This result is seen in Figure 9.

**Figure 10:**
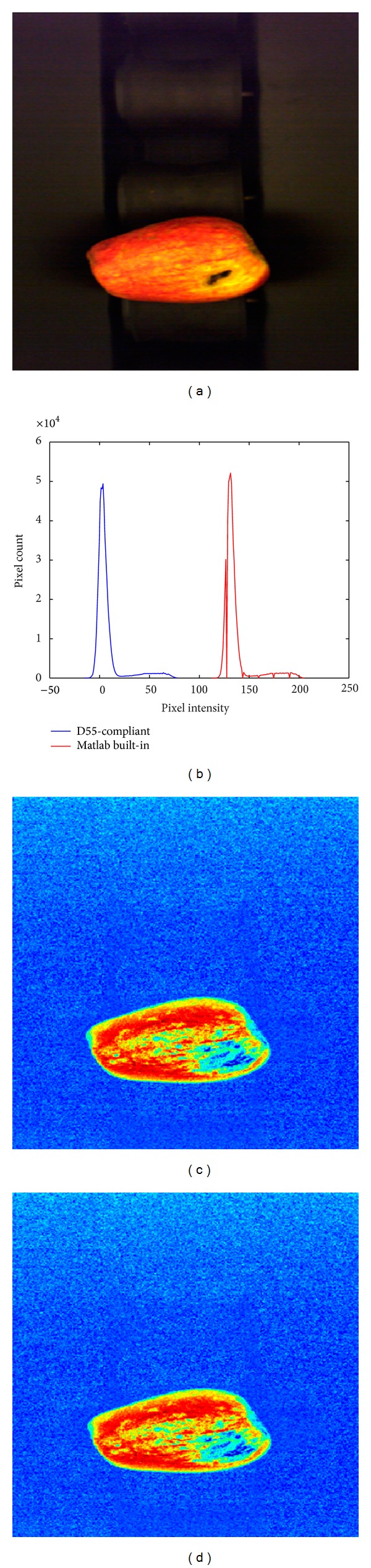
CIE L*a*b*/*a* component transform results; (a) original image; (b) histogram comparison (c); transform results according to D55-compliant program; (d) transform results according to matlab built-in rgb2lab function.

These two functions (D55-compliant and Matlab built-in) are implemented on RGB original image (Figure 10(a)) in order to obtain “*a*” component with red color sensitive to the CIE L*a*b* space. Comparing histograms obtained by two different functions, similarities are seen between aggregation curves of pixels, despite the different positions of histograms (Figure 10(b)). So the color space transformation of the methods gave similar results (Figures 10(c) and 10(d)).

As a result, 595 apples including three kinds, “Starking, Jonagold, and Golden”, were examined by the system (Table 2). They were classified into four categories according to their color and size. Finally, it was determined that success rate of the system was 99%.

Speed is yet another critical aspect to consider. With the use of a custom and fast D55-compliant program, 95 apples are classified in less than a minute, but the processing time to classify apples with the developed software according to two parameters like size and color is approximately 260 ms. Therefore, the speed of automatic apple classification system can be further increased with the improvement of the sorting unit.

## 4. Conclusions

Some important outputs drawn from the study on the apple classification system offered are as follows. In apple classification systems, there have been some important things as the follows: (i) what kind of apples is classified, (ii) which properties are considered for the classification, and (iii) what amount of apples are classified at a given time. Having such information indicates how much the system is practicable.

Examining these kinds of studies existing in the literature, it is seen that classifications were performed with respect to mainly two parameters like the segmentation of the surface images and the surface quality. In this study, classification of apples are practically realized considering the parameters, color, and size. For this, software based color space, instead of cameras which are sensitive to the different wavelengths and filters, is utilized to have the color and size information of apples. The different color spaces were compared and the most suitable ones were selected. In the determination of the color spaces, the cameras and lighting equipment used in the system have a great importance.

In this study, in accordance with the studies in the literature, a system which places under the camera and also which lets the camera make rotary motion was offered. With such a system, multiple photos can be taken and most of the surface (of the apple) can be viewed. Otherwise, naturally processing time increases because more photos are taken. To overcome this problem, only one photo was considered to get information about the color and size of the apples.

In this experimental study, the system works on only one line. If the number of the lines is increased, more apples can be classified by the system. Since the separator of the system works in a short distance, it can not keep up with the processing rate of the software. Though the classification rate of the software for an apple is 260 ms, which corresponds to 240 apples in one minute, the number of apples in a minute, in fact, remains 96. This problem can be solved easily by using a handle belt conveyor.

## Figures and Tables

**Table 1:** Experimental results of different color spaces for apples.

Color spaces	*R*	*G*	*B*	*H*	*S*	*V*
Red	0.099 ∓ 0.040	0.009 ∓ 0.004	0.010 ∓ 0.00	0.622 ∓ 0.314	0.750 ∓ 0.055	84.796 ∓ 17.059
Yellow	0.347 ∓ 0.128	0.288 ∓ 0.108	0.010 ∓ 0.004	0.154 ∓ 0.012	0.832 ∓ 0.043	153.669 ∓ 27.267
Background	0.013 ∓ 0.004	0.011 ∓ 0.003	0.023 ∓ 0.006	0.610 ∓ 0.142	0.307 ∓ 0.124	37.478 ∓ 5.902

Color spaces	*L*	*a*	*b*	*X*	*Y*	*Z*

Red	18.401 ∓ 4.154	29.095 ∓ 5.810	14.645 ∓ 5.22	0.046 ∓ 0.017	0.029 ∓ 0.011	0.013 ∓ 0.004
Yellow	57.922 ∓ 9.869	−4.756 ∓ 2.787	56.835 ∓ 8.805	0.248 ∓ 0.091	0.271 ∓ 0.107	0.051 ∓ 0.018
Background	9.894 ∓ 2.349	3.278 ∓ 1.660	−7.244 ∓ 2.793	0.013 ∓ 0.003	0.014 ∓ 0.004	0.023 ∓ 0.006

**Table 2:** Apple classification results.

Groups	Red/regular	Red/large	Yellow/regular	Yellow/large	Total
Graded	142	138	153	162	595
Incorrect classification	2	1	0	1	4
Correct classification rate	0.986	0.992	1	0.993	
Global correct classification rate		0.993			
